# Correction: Del Barrio-Galán, R., et al. Evaluation of Yeast Derivative Products Developed as an Alternative to Lees: The Effect on the Polysaccharide, Phenolic and Volatile Content, and Colour and Astringency of Red Wines. *Molecules* 2019, *24*, 1478

**DOI:** 10.3390/molecules24132385

**Published:** 2019-06-27

**Authors:** Rubén Del Barrio-Galán, Cristina Úbeda, Mariona Gil, Marcela Medel-Marabolí, Nathalie Sieczkowski, Álvaro Peña-Neira

**Affiliations:** 1 Department of Agro-Industry and Enology, Faculty of Agronomical Sciences, University of Chile, P.O. Box 1004, Santa Rosa 11315, La Pintana, Santiago, Chile; mmedel@uchile.cl (M.M.-M.); apena@uchile.cl (Á.P.-N.); 2 Lallemand Inc. Chile y Compañía Limitada, Rosario Norte 407, piso 6, Las Condes, Santiago, Chile; 3 Instituto de Ciencias Biomédicas, Facultad de Ciencias, Universidad Autónoma de Chile, Santiago 7500912, Chile; c_ubeda@us.es; 4 Instituto de Ciencias Químicas Aplicadas, Inorganic Chemistry and Molecular Materials Center, Universidad Autónoma de Chile, el Llano Subercaseaux 2801, San Miguel, Santiago, Chile; marionagilicortiella@gmail.com; 5 Lallemand SAS, 19 rue des Briquetiers, BP 59, 31 702 Blagnac, France; nsieczkowski@lallemand.com

The authors wish to make the following corrections to this paper [1]:

We would like to change the affiliation of one of the authors, Dr. Mariona Gil, from:

Mariona Gil ^1,4^

^1^ Department of Agro-Industry and Enology, Faculty of Agronomical Sciences, University of Chile, P.O. Box 1004, Santa Rosa 11315, La Pintana, Santiago, Chile

^4^ Instituto de Ciencias Químicas Aplicadas, Inorganic Chemistry and Molecular Materials Center, Universidad Autónoma de Chile, el Llano Subercaseaux 2801, San Miguel, Santiago, Chile

to the correct version, as follows:

Mariona Gil ^4^

^4^ Instituto de Ciencias Químicas Aplicadas, Inorganic Chemistry and Molecular Material Center, Facultad de Ingeniería, Universidad Autónoma de Chile,. Av. El Llano Subercaseaux 2801. San Miguel, Santiago, Chile

The authors would like to apologize for any inconvenience caused to the readers by these changes.
